# Fatigue in Multiple Sclerosis Is Associated With Childhood Adversities

**DOI:** 10.3389/fpsyt.2020.00811

**Published:** 2020-08-28

**Authors:** Gesa E. A. Pust, Christian Dettmers, Jennifer Randerath, Anne C. Rahn, Christoph Heesen, Roger Schmidt, Stefan M. Gold

**Affiliations:** ^1^Institute of Neuroimmunology and Multiple Sclerosis (INIMS), University Medical Center Hamburg-Eppendorf, Hamburg, Germany; ^2^Department of Psychology, University of Konstanz, Konstanz, Germany; ^3^ZIST, Penzberg, Germany; ^4^Lurija Institute for Rehabilitation and Health Sciences at the University of Konstanz, Schmieder Foundation for Sciences and Research, Allensbach, Germany; ^5^Department of Neurology, University Medical Centre Hamburg-Eppendorf (UKE), Hamburg, Germany; ^6^Klinik für Psychosomatik und Konsiliarpsychiatrie, Departement Innere Medizin, Kantonsspital St. Gallen, St. Gallen, Switzerland; ^7^Charité - Universitätsmedizin Berlin, Department of Psychiatry and Psychotherapy, Campus Benjamin Franklin, Berlin, Germany; ^8^Charité - Universitätsmedizin Berlin, Department of Psychosomatic Medicine, Campus Benjamin Franklin, Berlin, Germany

**Keywords:** fatigue, Multiple Sclerosis, childhood adversities, trait characteristics, psychopathology, path model

## Abstract

Fatigue is a common and disabling symptom in patients with Multiple Sclerosis (PwMS). Its pathogenesis, however, is still not fully understood. Potential psychological roots, in particular, have received little attention to date. The present study examined the association of childhood adversities, specific trait characteristics, and MS disease characteristics with fatigue symptoms utilizing path analysis. Five hundred and seventy-one PwMS participated in an online survey. Standardized psychometric tools were applied. The Childhood Trauma Questionnaire (CTQ) served to assess childhood adversities. Trait variables were alexithymia (Toronto Alexithymia Scale; TAS-26) and early maladaptive schemas (Young Schema Questionnaire; YSQ). Current pathology comprised depression (Beck’s Depression Inventory FastScreen; BDI-FS) and anxiety symptoms (State-Trait Anxiety Inventory; STAI-state), as well as physical disability (Patient determined Disease Steps; PDDS). The Fatigue Scale for Motor and Cognitive Functions (FSMC) was the primary outcome variable measuring fatigue. PwMS displayed high levels of fatigue and depression (mean FSMC score: 72; mean BDI-II score: 18). The final path model revealed that CTQ emotional neglect and emotional abuse remained as the only significant childhood adversity variables associated with fatigue. There were differential associations for the trait variables and current pathology: TAS-26, the YSQ domain impaired autonomy and performance, as well as all current pathology measures had direct effects on fatigue symptoms, accounting for 28.2% of the FSMC variance. Bayesian estimation also revealed indirect effects from the two CTQ subscales on FSMC. The final model fitted the data well, also after a cross-validation check and after replacing the FSMC with the Chalder Fatigue Questionnaire (CFQ). This study suggests an association psychological factors on fatigue in Multiple Sclerosis. Childhood adversities, as well as specific trait characteristics, seem to be associated with current pathology and fatigue symptoms. The article discusses potential implications and limitations.

## Introduction

Fatigue is among the most disabling symptom in patients with multiple sclerosis (PwMS) (1). It affects over 75% of all PwMS (2, 3). Moreover, fatigue is the primary factor for MS-related early retirement (4, 5). Research on the pathogenesis of MS fatigue has identified several (neuro-) biological, immunological, and neurophysiological correlates (1). However, the multifactorial genesis of MS-fatigue remains incompletely understood.

Several studies have suggested that psychological factors might also contribute to MS fatigue, as, for example, indicated by its association with depression (6, 7). Moreover, since behavioral interventions such as Cognitive Behavioral Therapy (CBT) or mindfulness-based approaches are capable of effectively reducing fatigue severity in this PwMS (8), psychological underpinnings appear to modulate aspects of fatigue too.

However, knowledge about the relative contribution of psychological factors to MS fatigue and their functional significance remain limited. Interestingly, evidence from other chronic disorders such as HIV (9, 10), cancer (11, 12), or chronic fatigue syndrome [CFS; (13, 14)] suggests a potential link between fatigue, early life adversities, and traumatic stress. The present exploratory study aimed to interrelate these factors, and assess their relative contribution to fatigue severity in a large sample of PwMS, by conducting an online survey and utilizing a path-analytic approach.

## Material and Methods

### Participants

Participants were recruited *via* advertisements on the website of the German MS Society (Deutsche Multiple Sklerose Gesellschaft, DMSG) from July 2018 to March 2019. In addition, flyers and newsletters were used to advertise the study at a large rehabilitation center (Kliniken Schmieder Konstanz) and the MS outpatient clinic of the University Medical Center Hamburg-Eppendorf.

Patients were eligible if they had a self-reported diagnosis of MS and if they were at least 18 years old. Access to the Internet was mandatory. Out of a total of 1.490 individuals who registered *via* the website, 608 (41%) completed the study. In order to avoid the imputation of missing data, only complete data sets were included (n = 571). All participants provided full informed consent prior to enrolment. The ethical review board of the University of Konstanz approved the study in June 2018, prior to enrolment of the first participant. The study was conducted following the European data protection regulation (EU-GDPR).

### Study Procedure

The flyers in the Kliniken Schmieder Konstanz, the University Medical Center Hamburg-Eppendorf, and the website of the DMSG provided a link to connect directly to the online survey (Unipark survey software, Globalpark AG, Hürth). PwMS provided informed consent online before participation. First, PwMS completed questions related demographic and clinical characteristics {sex, age, MS duration, medication, education, marital status, psychiatric disorders, and the Patient determined Disease Steps [PDDS; (15)] as a measure of disability}. Second, they completed a battery of questionnaires (see below). The duration of the whole survey was approximately 65 min.

### Measures

The study utilized two measures of fatigue, the *Fatigue Scale for Motor and Cognitive Functions* (FSMC), and the *Chalder Fatigue Questionnaire* [CFQ, (16)], using the validated German versions (17, 18). The German version of the *Beck Depression Inventory* [*BDI-II*, (19)] served to assess current symptoms of depression. A score of 13 is the cut-off score for as validated for the German version [see (20)] and has also been validated for use in pwMS (21, 22). For the inclusion into the path model, the shortened *BDI-II-FastScreen* was used [*BDI-FS*; (23)]. The BDI-FS omits items covering vegetative and somatic aspects of depression, thus avoiding confounding of the associations between depression and fatigue in the model that could be caused by overlapping items on fatigue and related symptoms. The German version of the *Patient-Determined Disease Steps* [*PDDS*, (15)] served as a measure of disability. The PDDS is a self-report measure which strongly correlates with the neurologist-rated Expanded Disability Status Scale [EDSS, (24)]. To assess alexithymia, the German version of the *Toronto-Alexithymia-Scale-26* [*TAS-26*, (25, 26)] was administered. The German version of the *Childhood Trauma Questionnaire* [CTQ, (27, 28)] is a self-report questionnaire for the assessment of adverse childhood experiences. It is valid for the application in individuals aged 12 years or older. The CTQ has five subscales that were all considered in the present study: 1. *CTQ emotional abuse*; 2. *CTQ physical abuse*; 3. *CTQ sexual abuse*; 4. *CTQ emotional neglect*; and 5. *CTQ physical neglect*. For the assessment of symptoms of anxiety, the present study used the short form of the German *State-Trait Anxiety Inventory* [STAI, (29, 30)], with an emphasis on the current symptoms, assessed with the State Anxiety Scale (*STAI-State)*. The *Young Schema-Questionnaire – Short Form* 3 [*YSQ*, (31)] is a measure to assess early maladaptive schemas (EMS), which has been translated and validated in German (32). Young (33) suggest that schemas form domains, which represent the hypothesized developmental origins of the schemas. Each of the domains represents a grouping of developmental needs. Young postulates five domains that were also considered in the present study: 1. *YSQ disconnection and rejection*; 2. *YSQ impaired autonomy and performance*; 3. *YSQ impaired limits*; 4. *YSQ other-directedness*; and 5. *YSQ over-vigilance and inhibition*. Detailed information on the scoring of each measure can be found in **Supplement 3**.

### Data Analysis

In a first step, Pearson-correlations between the main variables of interest included in the path analysis served to display the zero-order correlations. According to Cohen (34), correlation coefficients >.10 represent small, >.30 medium, and >.50 large effects. The supplement contains the respective table (**Supplement 1, 2**).

Path analyses calculated in AMOS 25 for Windows served to model the complex relations between 1) childhood adversities, 2) schema domains and alexithymia, 3) current pathology, and 4) fatigue. All other analyses were carried out with SPSS 25 for Windows. Fatigue, as measured with the FSMC, was the primary dependent variable. All other variables served as predictor variables on the respective levels:

The model followed the hypotheses that childhood precedes the development of a stable personality and that the development of a stable personality precedes current pathology and fatigue symptoms. Therefore, 1. CTQ subscales constituted the first level of the predictor variables, 2. YSQ schema domains and alexithymia the second level, and 3. the current depression and anxiety symptoms, as well as the subjectively reported disability, the third level. Statistical modeling started with the full model and a backward exclusion approach, excluding non-significant paths stepwise. During this procedure, one step also acknowledged the content of the relationship between variables where the identification of a non-significant path was equivocal due to a significant variance overlap between path-coefficients from two CTQ subscales. The fit indices CFI (comparative fit index) and RMSEA (root mean square error of approximation) according to the cut-off scores defined in Hu & Bentler (35) served to estimate the fit of the final model.

First, maximum likelihood estimation was applied. However, multivariate kurtosis and skewness values indicated deviations from multivariate normality [kurtosis = 10.16; c.r. = 7.18; (34)], in particular, due to skewed CTQ distributions. These deviations are critical as violations of multivariate normality may cause an inflation of Chi-Square values (35). The Bollen-Stine bootstrap *p* confirmed that the path model based on Maximum Likelihood estimation did not fit the data adequately (*p* = .021). Therefore, the repeated statistical modeling utilized the backward procedure with a bootstrapping approach. Bootstrapping, with 50.000 bootstrap samples, served to calculate bias-corrected confidence intervals for the path coefficients. Path coefficients that lay within the confidence interval ranging from (*SM* - 1,96*SE) to (*SM* + 1,96*SE) were considered as statistically not significant. In the final model, four cases displayed Mahalanobis distances at *p* <.001 fulfilling the criterion of outliers (36). However, the exclusion of these outliers did not have a significant impact on the final model. Thus, maintaining these data in the data set was justified.

Additionally, to support the validity of the final selected model, the model fit was re-estimated by using the second fatigue measure (CFQ) as the dependent variable. Furthermore, a cross-validation approach helped to estimate the robustness of the proposed model by randomly splitting the sample into two halves. Model fits were re-estimated for the split sub-samples. Finally, Bayesian estimation served to calculate the indirect effects of childhood adversities on fatigue symptoms. The Markov Chain Monte Carlo (MCMC) method implemented in AMOS was chosen. This Monte Carlo technique utilizes a random number generator to draw samples, serving to estimate the posterior distribution of parameters in the model. Ninety-five percent confidence intervals were chosen to determine statistical significance. The level of significance was alpha <.05.

## Results

Sample Characteristics **Table 1** provides an overview of the sample characteristics. Means (*SD*) represent the primary estimates unless otherwise specified.

**Table 1:** Sample characteristics.

	Total (*N* = 571)
**age in years** (*M/SD*)	43.4 (10.9)
**sex** (females/males)	438/133
**education**	
no graduation	2 (.4%)
9 years	39 (6.8%)
10 years	173 (30.3%)
13 years	357 (62.5%)
**disease duration in years** (*M/SD*)	9.1 (7.6)
**PDDS** (*M*/*SD*)	2.4 (1.9)
**Disease course** *N* (%)	
Clinically isolated syndrome	7 (1.2%)
Relapsing-remitting (RRMS)	392 (68.7%)
Secondary progressive (SPMS)	75 (13.1%)
Primary progressive (PPMS)	52 (9.1%)
Unknown	45 (7.9%)
**FSMC** (*M*/*SD*)	72.26 (16.16)
**CFQ** (*M*/*SD*)	21.06 (6.08)
**BDI-II** (*M*/*SD*)	17.71 (10.39)
**BDI-FS** (*M*/*SD*)	4.41 (3.63)
**Clinically relevant depressive symptoms** (BDI-II 13 or higher) N, %	374 (65.5)

N, number of participants; SD, Standard Deviation; M, mean; CFQ, Chalder Fatigue Questionnaire; FSMC, Fatigue Scale for Motor and Cognitive Functions; BDI-FS, Beck Depression Inventory II FastScreen; BDI-II, Beck Depression Inventory II; PDDS, Patient Determined Disease Steps.

The sample had a distribution of clinical characteristics reasonably typical for an MS outpatient sample with a female to male ratio of 3.3 to 1 and approximately 70% RRMS patients. However, depression scores were higher than what is typical for this population (65.5% above the clinical cut-off of ≥13 on the BDI-II for at least mild depressive symptoms).

### Path Model

Before inclusion in the path model, inter-correlations between the primary outcome variables were calculated. **Supplement 1** displays Pearson correlation coefficients between the fatigue measures and the measures assessing symptom severity. **Supplement 2** contains Pearson correlations between current symptom severity, fatigue, and childhood adversities, as well as schema domains (EMS) and alexithymia.

**Figure 1:**
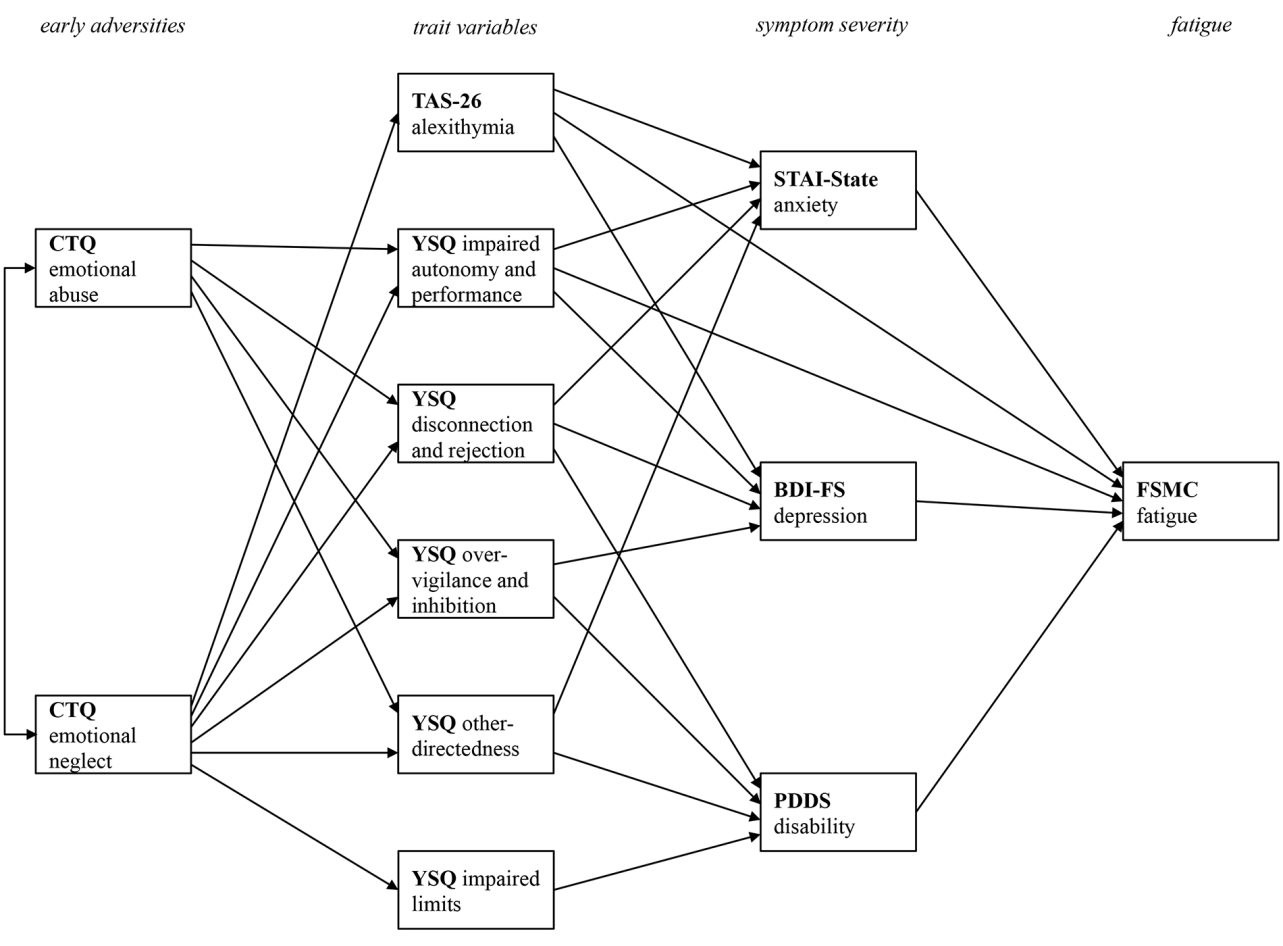
Final path model with all statistically significant paths between the four levels of early adversities, trait variables, current pathology, and fatigue symptoms. CTQ, Childhood Trauma Questionnaire; TAS-26, Toronto Alexithymia Scale 26; YSQ, Young Schema Questionnaire S3; STAI-State, State Trait Anxiety Inventory-State Scale; BDI-FS, Beck Depression Inventory II FastScreen; PDDS, Patient Determined Disease Steps; FSMC, Fatigue Scale for Motor and Cognitive Functions. Only statistically significant paths are printed.

In contrast to a multiple linear regression analyses that primarily focuses on the relationship between one dependent variable and various predictor variables, a path model has the advantage of combining different simple regression models into one overarching model. **Figure 1** displays the final path model after stepwise removal of non-significant paths in order to improve model fit. By removing non-significant paths from the initial model, only those paths that are meaningful in terms of the prediction of one variable from other variables remain in the final model.

The model fit for the final model was excellent [Chi^2^ (20) = 35.94, *p* = .016, Chi^2^/df = 1.80, CFI = 1.00, RMSEA = .04]. To facilitate the presentation of the complex relations between the variables, **Figure 1** only displays the relevant paths without the beta coefficient values. The subsequent figures focus on specific relationships. They also display the bootstrapped 95% confidence intervals.

**Figure 2:**
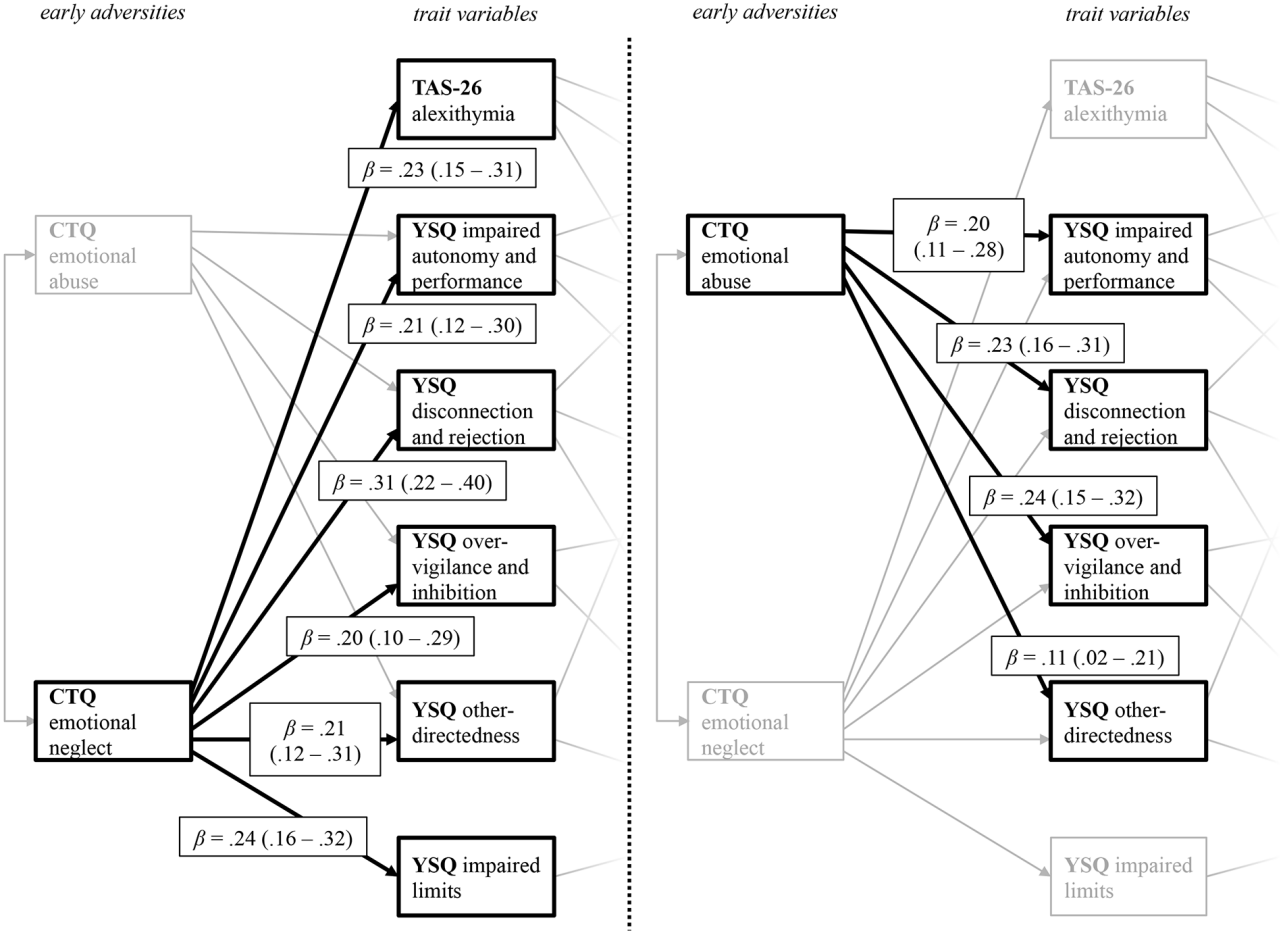
Path coefficients from the predictor variables CTQ emotional neglect (left) and CTQ emotional abuse (right) on the YSQ sub scales and the TAS 26 alexithymia scales. The figure displays the levels of early adversities and trait variables taken from the final path model displayed in **Figure 1**. CTQ, Childhood Trauma Questionnaire; TAS-26, Toronto Alexithymia Scale 26; YSQ, Young Schema Questionnaire S3. For a better visual presentation, the paths from the CTQ subscales *CTQ emotional neglect* (left) and *CTQ emotional abuse* (right) on the trait variables are printed separately, with paths belonging to the other predictor variable respectively being greyed out. Standardized beta coefficients are displayed.

**Figure 2** displays the relation between childhood adversities and trait measures, i.e., the first two levels of the path model. From the five different CTQ scales initially included in the full model, only emotional neglect and emotional abuse remained as significant predictors in the final model. For a better visual representation, **Figure 2** displays the path coefficient from the predictor variables CTQ emotional abuse and CTQ emotional neglect on the PwMSs’ trait characteristics, indicated by their EMSs (YSQ) and their level of alexithymia (TAS-26) separately. The results highlight the importance of emotional disturbances in childhood and adolescence. Both CTQ scales significantly predict four of the five YSQ scales (1. YSQ disconnection and rejection; 2. YSQ impaired autonomy and performance; 3. YSQ other-directedness; and 4. YSQ over-vigilance and inhibition) that all remained in the final model. Also, CTQ emotional neglect predicted alexithymia, and the YSQ subscale impaired limits.

**Figure 3:**
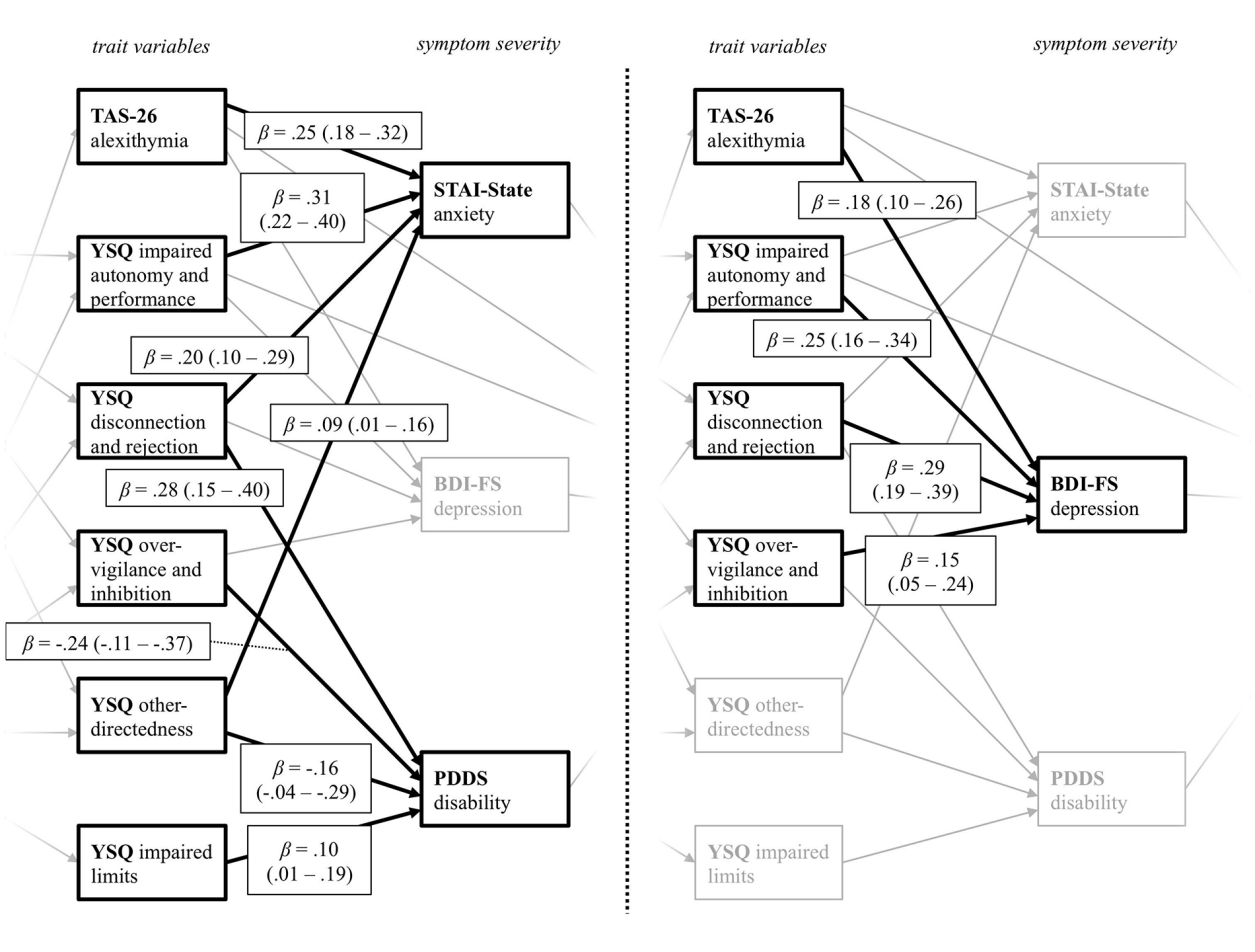
Path coefficients from the YSQ and TAS-26 trait variables on the variables assessing current symptom severity. The figure displays the levels of trait variables and symptom severity taken from the final path model displayed in **Figure 1**. YSQ, Young Schema Questionnaire S3; TAS-26, Toronto Alexithymia Scale 26; STAI-State, State Trait Anxiety Inventory State Scale; BDI-FS, Beck Depression Inventory II FastScreen; PDDS, Patient Determined Disease Steps. For a better visual presentation, the paths from the trait variables on the variables measuring current symptom severity *STAI-State* and *PDDS* (left), as well as the *BDI-FS* (right) are printed separately, with paths belonging to the other predictor variable respectively being greyed out. Standardized beta coefficients are displayed.

For the relation between the trait variables and current symptom severity (**Figure 3**), alexithymia (TAS-26) and the YSQ scales impaired autonomy and performance, disconnection and rejection, as well as over-vigilance and inhibition, predicted depression symptoms. Higher alexithymia scores, as well as higher perceived disconnection and rejection, higher impairments in autonomy and performance, and higher other-directedness predicted more severe anxiety symptoms. PwMS who reported higher levels of disconnection and rejection, as well as impaired limits, also scored higher on the PDDS scale. As indicated by the negative path coefficients, PwMS with lower other-directedness and less over-vigilance and inhibition reported higher disability (PDDS). These results indicate that specific trait characteristics, which in turn are predicted by early life adversities, do have not only a significant impact on the current anxiety and depression severity but are also associated with self-reported disability.

**Figure 4:**
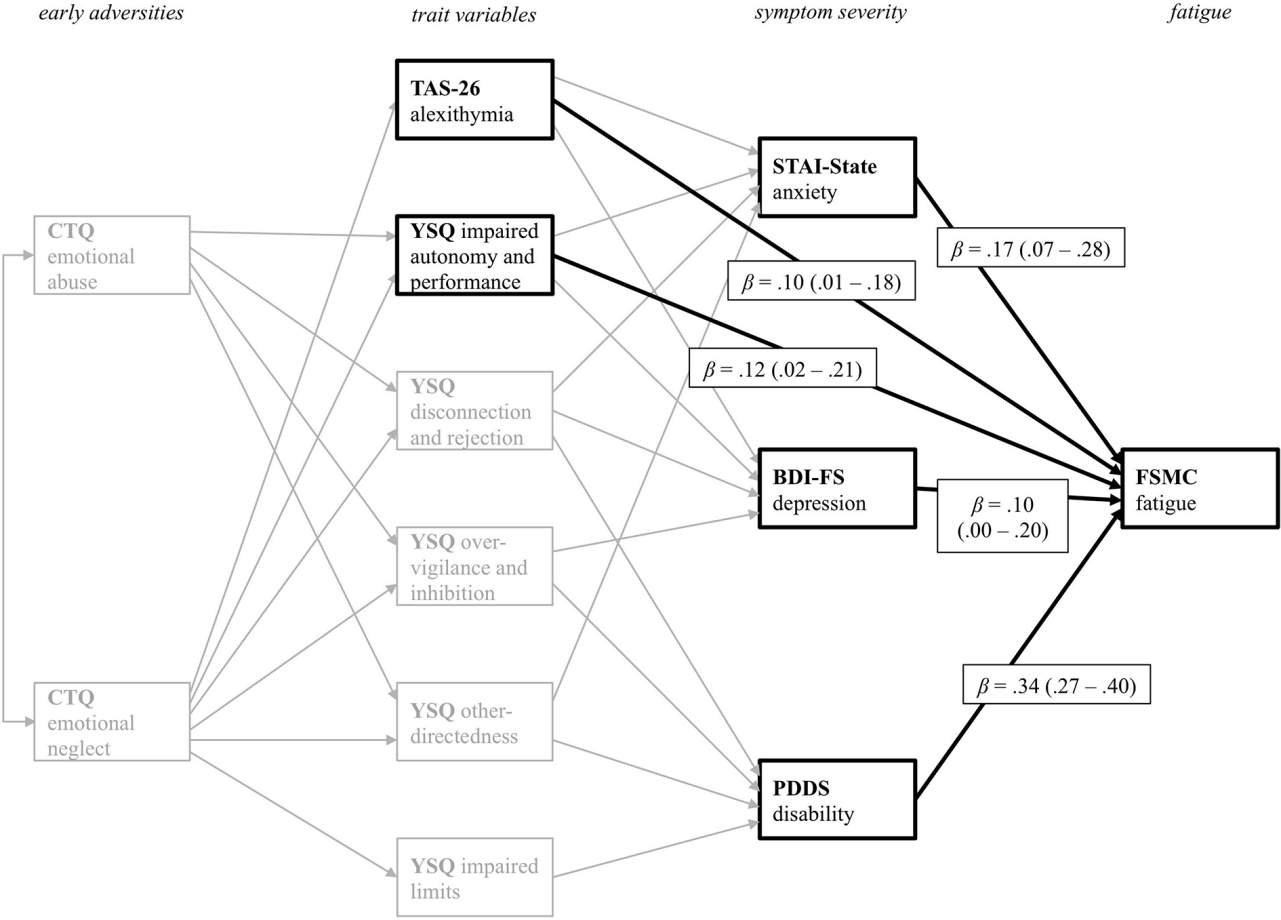
Path coefficients from all variables with direct effect on fatigue symptoms as the primary outcome variable *PDDS*. CTQ, Childhood Trauma Questionnaire; TAS-26, Toronto Alexithymia Scale 26; YSQ, Young Schema Questionnaire S3; STAI-State, State Trait Anxiety Inventory State Scale; BDI-FS, Beck Depression Inventory II FastScreen; PDDS, Patient Determined Disease Steps; FSMC, Fatigue Scale for Motor and Cognitive Functions. For a better visual presentation, only significant direct paths from the various predictor variables on the *FSMC* primary outcome variable are printed in black. Standardized beta coefficients are displayed.

**Figure 4** displays all direct significant predictor variables for the prediction of current fatigue severity. Current anxiety and depressive symptom severity directly influence fatigue severity. More severe symptoms of anxiety and depression are associated with higher fatigue scores. PwMS with higher disability also reported more severe fatigue symptoms. As indicated by the direct path from the TAS-26, PwMS with higher scores in alexithymia tend to experience higher fatigue severity. Additionally, PwMS who report impaired autonomy and performance on the respective YSQ-sub-scale also tend to report more fatigue symptoms. Thus, the trait variables TAS-26 and YSQ impaired autonomy and performance, also have a direct impact on fatigue symptoms. Additionally, there were small but significant indirect effects of reported emotional abuse (*M* = .05, *SE* <.01) and emotional neglect (*M* = .11, *SE* <.01) on fatigue. Thus, adverse emotional experiences during childhood also indirectly influence current fatigue symptoms. The path model accounted for 28.2% of the fatigue severity variance.

### Validation of the Path Model With a Second Fatigue Measure

The model fit remained excellent after re-calculating the final model with the CFQ instead of the FSMC as a measure for fatigue [Chi^2^ (20) = 34.25, *p* = .024, Chi^2^/df = 1.71, CFI = 1.00, RMSEA = .04]. This result supports the validity of the obtained model, as it was possible to replicate it with another fatigue measure.

### Cross-Validation Check

As another approach to support the validity of the model, it was re-calculated after splitting the sample into two equally large halves. This method serves to test whether the fit measures remain stable, when a different composition of the sample is chosen, or whether the fit measures are susceptible to one particular selection of participants and not replicable. As for the previous calculations, data fit the hypothesized model in both subsamples [split half 1 (*n* = 285): Chi^2^ (20) = 21.86, *p* = .348, Chi^2^/df = 1.09, CFI = 1.00, RMSEA = .02; split half 2 (*n* = 286): Chi^2^ (20) = 32.78, *p* = .036, Chi^2^/df = 1.64, CFI = .99, RMSEA = .05]. Similar results were observed after replacing the FSMC with the CFQ [split half 1 (*n* = 285): Chi^2^ (20) = 21.84, *p* = .349, Chi^2^/df = 1.09, CFI = 1.00, RMSEA = .02; split half 2 (*n* = 286): Chi^2^ (20) = 30.06, *p* = .069, Chi^2^/df = 1.50, CFI = .99, RMSEA = .04]. Thus, the obtained results support the validity of the final model obtained.

## Discussion

The present study aimed to investigate the association of different psychological factors with current fatigue symptoms in MS. In particular, it focused on 1) childhood adversities as measures of early adverse experiences, 2) Alexithymia and EMS according to the Schema-therapeutic model as measures of trait characteristics, 3) anxiety, depression symptoms, as well as disability, and 4) fatigue as the primary outcome variable. Path analysis served to model the complex relations and the relative contributions of these variables to fatigue severity.

Among the five CTQ childhood adversities subscales, only the subscales assessing emotional neglect and abuse remained in the final model, while all other subscales made no significant contribution (physical abuse, sexual abuse, and physical neglect). This finding corroborates and extends a previous cohort study (37), where emotional abuse and emotional neglect showed the largest effect sizes among the different types of childhood trauma in MS compared to healthy controls. Together, this adds to a growing literature from cross-sectional (37, 38) and register-based studies (39) that have indicated a link between childhood adversity, trauma-related disorders, and autoimmune diseases. While the available data suggest some specificity of emotional vs. physical domains of abuse, the potential causes for this remain unknown. Some of this might be due to statistical power as emotional abuse tends to have a higher reported frequency in MS [e.g. (37)]. It is tempting to speculate that other factors, including possible differential biological substrates of different forms of abuse, could also play a role here and this should be investigated in future research.

Further studies should focus on the comparison between PwMS, patients with other somatic disorders and co-morbid fatigue, as well as healthy controls. This would help to disentangle the specificity of the link between adverse emotional experiences and fatigue as an MS-related or a more general phenomenon. A link between adverse emotional experiences and fatigue has also been reported in other medical conditions, such as CFS or cancer (40, 41).

It should be noted that these findings could at least in part be due to an overlap with comorbid or misdiagnosed depression (14). In the present study, we used the BDI-II-FastScreen to overcome the shortcoming of a symptom overlap (i.e. somatic/vegetative symptoms of depression that may also be impact responses on fatigue questionnaires). Still, the differentiation between the different phenomena requires further study.

While our study was a cross-sectional survey, the assessment of emotional neglect and emotional abuse using the CTQ is a retrospective assessment of experiences during childhood and adolescence. Thus, these experiences will in most or all cases be before MS onset (which only rarely manifests before the age of 18 and typically has an age of onset between 20 and 30 years of age). There are several possibilities that may explain the increased frequencies of adverse childhood experiences reported by MS patients compared to healthy controls in previous studies (37). For example, it is conceivable that MS patients are more likely to recall or report such experiences (a phenomenon known as “effort after meaning”). However, the large population-based by Song et al. (39) used documented diagnoses of stress-related disorders (including PTSD) preceding the MS diagnosis by several years and thus cannot be explained by “effort after meaning”. This may suggest that direct or indirect effects of childhood adversity may increase the risk of subsequently developing MS (or other autoimmune diseases). Currently these mechanisms remain unknown but they may include behavioral factors [e.g. altered health behaviors such as diet, exercise, or smoking, the latter of which is a known risk factor for MS, see (42)]. Moreover, there might be biological pathways (e.g. altered stress response system regulation or inflammatory mechanisms) that have been linked to childhood trauma (43) and might also play a role in MS (44). Similarly, these mechanisms could also contribute to MS symptoms such as fatigue. However, at this point, this remains speculative and detailed studies, ideally longitudinal in nature, are required to probe this hypothesis and explore potential behavioral or biological mechanisms that might contribute to the association between childhood adversity and MS risk or symptom severity.

Based on the present analysis, the reported childhood adversities may not only be more common in MS than the general population but could indirectly contribute to symptom severity, particularly concerning depression and fatigue. The presented model suggests that these associations could be mediated *via* Early Maladaptive Schemas as measured by the YSQ.

These results are in line with studies in which early adversities predicted the development of EMS in particular in affective disorders (45) and individuals with personality disorders (46). Similarly, research on different psychopathologies, such as affective disorders (47), personality disorders (48), or adult dissociation (49), has reported a relation between emotional abuse/neglect and the development of alexithymia as a trait characteristic. The MS literature also consistently describes moderate associations between symptoms of anxiety, depression as well as disability, and fatigue (50, 51), which the proposed path model from our study confirms. Intriguingly, trait characteristics predicted current self-reported disability, which may reflect an overlap between psychological and somatic phenomena. However, while the associations were statistically significant in this large cohort, the magnitude of the contribution of YSQ and alexithymia to fatigue scores was rather small and a sizeable portion of variability in fatigue severity remained unexplained, suggesting that other factors not included in our model likely contribute to MS-associated fatigue.

Considering the relation between adverse childhood experiences and MS-related fatigue indicated by our data, it is tempting to hypothesize that Schema Therapy could provide a useful taxonomy to understand and link the functional aspect of fatigue to childhood adversities. Schema Therapy provides a trans-diagnostic conceptual and empirically validated framework (32, 51). A central aspect of schema therapy is the concept of EMS, thought to develop in childhood (52). While this might be interesting from a conceptual point of view, the cross-sectional associations between schemas and fatigue alone are not sufficient to claim causality, and no clinical trial to date has explored the potential of schema therapy for neuropsychiatric aspects of MS (including fatigue).

However, further research is required in order to elaborate a profound etiologcal working model on how early life adversities could manifest in MS and contribute to associated symptoms such as fatigue or psychopathology. While Young’s Schema Questionnaire assesses EMS, it does not capture the functional or dysfunctional nature of these concepts. Measures, such as the OPD conflict questionnaire (53) or the Schema Mode Inventory (54) could deepen our understanding of the psychosomatic or psychodynamic nature of fatigue. Fatigue could for example constitute a potential defense mechanism according to psychodynamic theories. Moreover, interactions on both the psychological and the biological level could account for the specific relation between adverse emotional experience and MS-fatigue found in this study and should be targeted by future research strategies.

The major limitation of the present study is that all data were obtained cross-sectionally and are correlational and the path model’s hierarchical structure does not necessarily imply a cause-effect relationship between the three levels. Moreover, responses in the CTQ specifically may be prone to recall bias or effort-after-meaning effects.

The present study was web-based and all MS diagnoses and clinical data rely on participants’ self-reports A selection bias for the PwMS sample can also not be excluded. The advertisement of the study as a survey on fatigue in MS may explain the high average levels of fatigue in our sample. In a related matter, depression scores were quite high, and the percentage of patients who had depressive symptoms above the clinical cut-off was higher than what is common in MS (55). Structured clinical interviews would have been helpful to validate anxiety and depression symptoms. Bed-ridden patients or patients who do not have access to the internet might also not have participated. Biased sample characteristics could limit the generalizability of the present findings.

Moreover, MS has a genetic component and can cluster in families (56). Although the majority of MS patients do not have first-degree relatives with the disease, it is conceivable that genetic or environmental factors linked to family history of MS can affect childhood experiences. In our study, we did not assess and thus could not explore the effect of parental MS on any of the measures obtained.

Taken together, the present study revealed a path model based on a large sample of PwMS from an online-survey. Among different childhood adversities, only emotional neglect and emotional abuse predicted certain personality traits, current disability, and psychopathology, as well as MS fatigue symptoms. Alexithymia, as well as EMS, play a mediating role in the relation between childhood emotional abuse/neglect and current disability, as well as symptoms of depression and anxiety. Current disability and symptoms of depression and anxiety were, in turn, associated with fatigue symptoms. Thus, the current study not only confirms the well-established relation between psychopathology and fatigue in MS but is -to the best of our knowledge- the first to also establish a link to early adversities and specific trait characteristics. If early abuse and neglect increase the probability for the development of individual personality predispositions and the susceptibility to develop fatigue, this might provide a new perspective on the genesis of MS-fatigue.

## Data Availability Statement

The datasets generated for this study are available upon request to the corresponding author.

## Ethics Statement

The studies involving human participants were reviewed and approved by Ethical review board of the University of Konstanz. The patients/participants provided their written informed consent to participate in this study.

## Author Contributions

GP, RS, CH, and SG designed the study. GP, CD, and JR were responsible for data collection. GP conducted the statistical analysis and the data pre-processing. All authors interpreted the results and gave critical feedback. GP and SG wrote the manuscript. CD, JR, AR, CH, and RS critically revised the manuscript.

## Conflict of Interest

GP received speaker honoraria and project funding from Genzyme Sanofi and speaker honoraria from Novartis. CD has received honoraria from Novartis and Merck. CH received travel and speaker honoraria as well as research grants from Celgene, Genzyme, Merck, Novartis, Serono. RS reports speaker honoraria from Novartis. SG reports honoraria from Mylan GmbH, Almirall S. A., and Celgene and research grants from Biogen.

The remaining authors declare that the research was conducted in the absence of any commercial or financial relationships that could be construed as a potential conflict of interest.

## References

[1] Penner IK Paul F Fatigue as a symptom or comorbidity of neurological diseases. Nat Rev Neurol (2017) 13(11):662. 10.1038/nrneurol.2017.117 29027539

[2] Fisk JD Pontefract A Ritvo PG Archibald CJ Murray TJ The impact of fatigue on patients with multiple sclerosis. Can J Neurol Sci (1994) 21(1):9–14. 10.1017/S0317167100048691 8180914

[3] Hadjimichael O Vollmer T Oleen-Burkey M Fatigue characteristics in multiple sclerosis: the North American Research Committee on Multiple Sclerosis (NARCOMS) survey. Health Qual Life Outcomes (2008) 6(1):100. 10.1186/1477-7525-6-100 19014588 PMC2596785

[4] Gerhard L Dorstyn DS Murphy G Roberts RM Neurological, physical and sociodemographic correlates of employment in multiple sclerosis: a meta-analysis. J Health Psychol (2018) 95:92–104. 10.1177/1359105318755262 29460636

[5] Kobelt G Thompson A Berg J Gannedahl M Eriksson J MSCOI Study Group New insights into the burden and costs of multiple sclerosis in Europe. Multiple Sclerosis J (2017) 23(8):1123–36. 10.1177/1352458517694432 PMC5476197 28273775

[6] Chwastiak LA Gibbons LE Ehde DM Sullivan M Bowen JD Bombardier CH Fatigue and psychiatric illness in a large community sample of persons with multiple sclerosis. J Psychosom Res (2005) 59(5):291–8. 10.1016/j.jpsychores.2005.06.001 16253619

[7] Weinges-Evers N Brandt AU Bock M Pfueller CF Dörr J Bellmann-Strobl J Correlation of self-assessed fatigue and alertness in multiple sclerosis. Multiple Sclerosis J (2010) 16(9):1134–40. 10.1177/1352458510374202 20610494

[8] Wendebourg MJ Heesen C Finlayson M Meyer B Pöttgen J Köpke S Patient education for people with multiple sclerosis-associated fatigue: A systematic review. PloS One (2017) 12(3):e0173025. 10.1371/journal.pone.0173025 28267811 PMC5340368

[9] Barroso J Hammill BG Leserman J Salahuddin N Harmon JL Pence BW Physiological and psychosocial factors that predict HIV-related fatigue. AIDS Behav (2010) 14(6):1415–27. 10.1007/s10461-010-9691-2 PMC2975810 20352317

[10] Barroso J Leserman J Harmon JL Hammill B Pence BW Fatigue in HIV-infected people: a three-year observational study. J Pain Symptom Manage (2015) 50(1):69–79. 10.1016/j.jpainsymman.2015.02.006 25701691 PMC4492863

[11] Bower JE Crosswell AD Slavich GM Childhood adversity and cumulative life stress: risk factors for cancer-related fatigue. Clin Psychol Sci (2014) 2(1):108–15. 10.1177/2167702613496243 PMC3873097 24377083

[12] Han TJ Felger JC Lee A Mister D Miller AH Torres MA Association of childhood trauma with fatigue, depression, stress, and inflammation in breast cancer patients undergoing radiotherapy. Psycho-Oncology (2016) 25(2):187–93. 10.1002/pon.3831 PMC4973143 25976322

[13] Kempke S Luyten P De Coninck S Van Houdenhove B Mayes LC Claes S Effects of early childhood trauma on hypothalamic–pituitary–adrenal (HPA) axis function in patients with Chronic Fatigue Syndrome. Psychoneuroendocrinology (2015) 52:14–21. 10.1016/j.psyneuen.2014.10.027 25459889

[14] De Venter M Illegems J Van Royen R Moorkens G Sabbe BG Van Den Eede F Differential effects of childhood trauma subtypes on fatigue and physical functioning in chronic fatigue syndrome. Compr Psychiatry (2017) 78:76–82. 10.1016/j.comppsych.2017.07.006 28806608

[15] Learmonth YC Motl RW Sandroff BM Pula JH Cadavid D Validation of patient determined disease steps (PDDS) scale scores in persons with multiple sclerosis. BMC Neurol (2013) 13(1):37. 10.1186/1471-2377-13-37 23617555 PMC3651716

[16] Chalder T Berelowitz G Pawlikowska T Watts L Wessely S Wright D Development of a fatigue scale. J Psychosom Res (1993) 37(2):147–53. 10.1016/0022-3999(93)90081-P 8463991

[17] Penner IK Raselli C Stöcklin M Opwis K Kappos L Calabrese P The Fatigue Scale for Motor and Cognitive Functions (FSMC): validation of a new instrument to assess multiple sclerosis-related fatigue. Multiple Sclerosis J (2009) 15(12):1509–17. 10.1177/1352458509348519 19995840

[18] Martin A Staufenbiel T Gaab J Rief W Brähler E Messung chronischer Erschöpfung–Teststatistische Prüfung der Fatigue Skala (FS). Z für klinische Psychol und Psychotherapie (2010) 39:33–44. 10.1026/1616-3443/a000010

[19] Kühner C Bürger C Keller F Hautzinger M Reliabilität und validität des revidierten beck-depressionsinventars (BDI-II). Der Nervenarzt (2007) 78(6):651–6. 10.1007/s00115-006-2098-7 16832698

[20] Schneider F Härter M Schorr S (Eds.) S3-Leitlinie/Nationale VersorgungsLeitlinie Unipolare Depression. Springer: Verlag (2017).

[21] Schippling S O’Connor P Knappertz V Pohl C Bogumil T Suarez G Incidence and course of depression in multiple sclerosis in the multinational BEYOND trial. J Neurol (2016) 263:1418–26. 10.1007/s00415-016-8146-8 PMC4929160 27177997

[22] Solaro C Gamberini G Masuccio FG Depression in multiple sclerosis: epidemiology, aetiology, diagnosis and treatment. CNS Drugs (2018) 32(2):117–33. 10.1007/s40263-018-0489-5 29417493

[23] Kliem S Mößle T Zenger M Brähler E Reliability and validity of the Beck Depression Inventory-Fast Screen for medical patients in the general German population. J Affect Disord (2014) 156:236–9. 10.1016/j.jad.2013.11.024 24480380

[24] Kurtzke JF Rating neurologic impairment in multiple sclerosis: an expanded disability status scale (EDSS). Neurology (1983) 33(11):1444–4. 10.1212/WNL.33.11.1444 6685237

[25] Taylor GJ Bagby RM Ryan DP Parker JD Validation of the alexithymia construct: a measurement-based approach. Can J Psychiatry (1990) 35(4):290–7. 10.1177/070674379003500402 2346893

[26] Kupfer J Brosig B Brähler E Toronto-Alexithymie-Skala-26: TAS-26; deutsche Version. Hogrefe: Göttingen (2001).

[27] Bernstein DP Stein JA Newcomb MD Walker E Pogge D Ahluvalia T Development and validation of a brief screening version of the Childhood Trauma Questionnaire. Child Abuse Negl (2003) 27(2):169–90. 10.1016/S0145-2134(02)00541-0 12615092

[28] Klinitzke G Romppel M Häuser W Brähler E Glaesmer H Die deutsche version des childhood trauma questionnaire (CTQ)–psychometrische Eigenschaften in einer bevölkerungsrepräsentativen Stichprobe. PPmP-Psychotherapie· Psychosomatik· Medizinische Psychol (2012) 62(02):47–51. 10.1055/s-0031-1295495 22203470

[29] Spielberger CD Gorsuch RL Lushene RE Stai. Manual for the State-Trait Anxiety Inventory (Self Evaluation Questionnaire) Vol. 22 Palo Alto California: Consulting Psychologist (1970) p. 1–24.

[30] Grimm J (Hg.) State-Trait-Anxiety Inventory nach Spielberger. Deutsche Lang- und Kurzversion. MF-Working Paper: Methodenforum der Universität Wien (2009). 2009/02.

[31] Bach B Simonsen E Christoffersen P Kriston L The Young Schema Questionnaire 3 Short Form (YSQ-S3). Eur J Psychol Assess (2015) 33:134–43. 10.1027/1015-5759/a000272

[32] Kriston L Schäfer J Jacob GA Härter M Hölzel LP Reliability and validity of the German version of the Young Schema Questionnaire–Short Form 3 (YSQ-S3). Eur J Psychol Assess (2013) 29:205–12. 10.1027/1015-5759/a000143

[33] Young JE Cognitive therapy for personality disorders: A schema-focused approach. Professional Resource Press: Sarasota (1999).

[34] Cohen J Statistical power analysis for the behavioral sciences. 2nd ed. Hillsdale, NJ: Erlbaum (1988).

[35] Hu LT Bentler PM Cutoff criteria for fit indexes in covariance structure analysis: conventional criteria versus new alternatives. Struct Equation Model: Multidiscip J (1999) 6(1):1–55. 10.1080/10705519909540118

[36] Kline RB Convergence of Structural Equation Modeling and Multilevel Modeling. In: Williams M Vogt WP, editors. The SAGE handbook of innovation in social research methods. Sage: Thousand Oaks (2011).

[37] Spitzer C Bouchain M Winkler LY Wingenfeld K Gold SM Grabe HJ Childhood trauma in multiple sclerosis: a case-control study. Psychosom Med (2012) 74(3):312–8. 10.1097/PSY.0b013e31824c2013 22408134

[38] Hellmann-Regen J Piber D Hinkelmann K Gold SM Heesen C Spitzer C Depressive syndromes in neurological disorders. Eur Arch Psychiatry Clin Neurosci (2013) 263(2):123–36. 10.1007/s00406-013-0448-6 24077889

[39] Song H Fang F Tomasson G Arnberg FK Mataix-Cols D de la Cruz LF Association of stress-related disorders with subsequent autoimmune disease. Jama (2018) 319(23):2388–400. 10.1001/jama.2018.7028 PMC6583688 29922828

[40] Kempke S Luyten P Claes S Van Wambeke P Bekaert P Goossens L The prevalence and impact of early childhood trauma in Chronic Fatigue Syndrome. J Psychiatr Res (2013) 47(5):664–9. 10.1016/j.jpsychires.2013.01.021 23421962

[41] Bower JE The role of neuro-immune interactions in cancer-related fatigue: Biobehavioral risk factors and mechanisms. Cancer (2019) 125(3):353–64. 10.1002/cncr.31790 PMC6502236 30602059

[42] Dengelmann ML Hermann KM Smoking and multiple sclerosis: a systematic review and meta-analysis using Bradford-Hill criteria for causation. Multiple Sclerosis Rel Disord (2017) 17:207–16. 10.1016/j.msard.2017.07.020 29055459

[43] Heim CM Entringer S Buss C Translating basic research knowledge on the biological embedding of early-life stress into novel approaches for the developmental programming of lifelong health. Psychoneuroendocrinology (2019) 105:123–37. 10.1016/j.psyneuen.2018.12.011 PMC6561839 30578047

[44] Gold SM Mohr DC Huitinga I Flachenecker P Sternberg EM Heesen C The role of stress-response systems for the pathogenesis and progression of MS. Trends Immunol (2005) 26(12):644–52. 10.1016/j.it.2005.09.010 16214415

[45] Lumley MN Harkness KL Specificity in the relations among childhood adversity, early maladaptive schemas, and symptom profiles in adolescent depression. Cogn Ther Res (2007) 31:639–57. 10.1007/s10608-006-9100-3

[46] Bach B Lockwood G Young JE A new look at the schema therapy model: organization and role of early maladaptive schemas. Cogn Behav Ther (2018) 47(4):328–49. 10.1080/16506073.2017.1410566 29256336

[47] Serafini G Gonda X Pompili M Rihmer Z Amore M Engel-Yeger B The relationship between sensory processing patterns, alexithymia, traumatic childhood experiences, and quality of life among patients with unipolar and bipolar disorders. Child Abuse Negl (2016) 62:39–50. 10.1016/j.chiabu.2016.09.013 27792883

[48] Deborde AS Miljkovitch R Roy C Dugré-Le Bigre C Pham-Scottez A Speranza M Alexithymia as a mediator between attachment and the development of borderline personality disorder in adolescence. J Pers Disord (2012) 26(5):676–88. 10.1521/pedi.2012.26.5.676 23013337

[49] Terock J Van der Auwera S Janowitz D Spitzer C Barnow S Miertsch M From childhood trauma to adult dissociation: the role of PTSD and alexithymia. Psychopathology (2016) 49(5):374–82. 10.1159/000449004 27623153

[50] Bol Y Duits AA Hupperts RM Vlaeyen JW Verhey FR The psychology of fatigue in patients with multiple sclerosis: a review. J Psychosom Res (2009) 66(1):3–11. 10.1016/j.jpsychores.2008.05.003 19073287

[51] Taylor CD Bee P Haddock G Does schema therapy change schemas and symptoms? A systematic review across mental health disorders. Psychol Psychother: Theory Res Pract (2017) 90(3):456–79. 10.1111/papt.12112 PMC5573974 28035734

[52] Young JE Klosko JS Weishaar ME Schema therapy. New York: Guilford (2003). p. 254.

[53] Benecke C Henkel M Doering S Jakobsen T Stasch M Dahlbender R Der OPD-Konfliktfragebogen. Z für Psychosomatische Med und Psychotherapie (2018) 64(4):380–93. 10.13109/zptm.2018.64.4.380 30829169

[54] Reiss N Dominiak P Harris D Knörnschild C Schouten E Jacob GA Reliability and validity of the German version of the Schema Mode Inventory. Eur J Psychol Assess (2012) 28:297–304. 10.1027/1015-5759/a000110

[55] Boeschoten RE Braamse AM Beekman AT Cuijpers P van Oppen P Dekker J Prevalence of depression and anxiety in multiple sclerosis: a systematic review and meta-analysis. J Neurol Sci (2017) 372:331–41. 10.1016/j.jns.2016.11.067 28017241

[56] Canto E Oksenberg JR Multiple sclerosis genetics. Multiple Sclerosis J (2018) 24(1):75–9. 10.1177/1352458517737371 29307290

