# Supplementary material for: Fatigue in Multiple Sclerosis Is Associated With Childhood Adversities
Source: Front Psychiatry. 2020 Aug 28;11:811. doi: 10.3389/fpsyt.2020.00811 (PMC7485280; doi:10.3389/fpsyt.2020.00811)
Supplement: Supplementary file 1 [file DataSheet_1.pdf]

Supplementary Material

Supplementary Material

Supplement 1

Supplement 1. Inter-correlations between the fatigue measures FSMC and CFQ, the BDI-II, BDI-FS, PDDS, and the STAI-State.

	FSMC	CFQ	BDI-II	BDI-FS	PDDS	STAI-State
FSMC	1					
CFQ	**.70****	1				
BDI-II	**.53****	**.60****	1			
BDI-FS	**.38****	**.46****	**.90****	1		
PDDS	**.35****	**.19****	**.11***	.07	1	
STAI-State	**.36****	**.42****	**.73****	**.70****	-.01	1

Note. ** $p < .001$; * $p < .01$. FSMC = Fatigue Scale for Motor and Cognitive Functions, CFQ = Chalder Fatigue Questionnaire, BDI-II = Beck Depression Inventory II, BDI-FS = Beck Depression Inventory II FastScreen, PDDS = Patient Determined Disease Steps, STAI-State = State Trait Anxiety Inventory State Scale. Significant correlation coefficients are printed bold.

Supplement 2

Supplement 2. Inter-correlations between the fatigue scales FSMC and CFQ, the BDI-II, BDI-FS, TAS-26, PDDS, STAI-State and the CTQ subscales as well as the YSQ schema-domains.

	FSMC	CFQ	BDI-II	BDI-FS	TAS-26	PDDS	STAI-State
CTQ emotional abuse	**.22****	**.20****	**.39****	**.36****	**.14****	.01	**.33****
CTQ physical abuse	**.12***	.07	**.18****	**.15****	.03	.01	**.14****
CTQ sexual abuse	.06	.05	.04	.01	-.03	-.03	.07
CTQ emotional neglect	**.23****	**.15****	**.38****	**.35****	**.23****	.08	**.35***
CTQ physical neglect	**.21****	**.15****	**.32****	**.27****	**.20****	**.15****	**.27****
YSQ disconnection and rejection	**.33****	**.34****	**.68****	**.68****	**.55****	.04	**.61****
YSQ impaired autonomy and performance	**.35****	**.37****	**.66****	**.66****	**.51****	.03	**.63****
YSQ impaired limits	**.24****	**.21****	**.43****	**.39****	**.37****	.02	**.39****
YSQ over-vigilance and inhibition	**.23****	**.28****	**.63****	**.63****	**.50****	**-.10**$^{+}$	**.57****
YSQ other-directedness	**.22****	**.25****	**.53****	**.50****	**.42****	**-.10**$^{+}$	**.50****

Note. ** $p < .001$; * $p < .01$. $^{+}p < .05$. CTQ = Childhood Trauma Questionnaire, YSQ = Young Schema Questionnaire S3, FSMC = Fatigue Scale for Motor and Cognitive Functions, CFQ = Chalder Fatigue Questionnaire, BDI-II = Beck Depression Inventory II, BDI-FS = Beck Depression Inventory II FastScreen, PDDS = Patient Determined Disease Steps, TAS-26 = Toronto Alexithymia Scale 26, STAI-State = State Trait Anxiety Inventory State Scale. Significant correlation coefficients are printed bold.

Supplement 3

Calculation of scale scores

Fatigue Scale for Motor and Cognitive Functions (FSMC)

The FSMC is a 20-item fatigue measure. Ten items measure symptoms of motor and cognitive fatigue, respectively [16]. PwMS had to score their responses on a five-point Likert scale (from “1” = absolutely agree” to “5” = “absolutely disagree”). The global FSMC score is the sum of the 20 item scores [range: 20-100]. The present study did not consider the two sub-scores for motor and cognitive fatigue.

Chalder Fatigue Questionnaire (CFQ)

The CFQ [17] is awell-established measure to assess patients’ fatigue severity. It includes 11 items referring to symptom severity in the past month. PwMS had to score their responses on a four-point Likert scale (“0” = “less than usual”, to “3” “much more than usual”) [18]. The analyses in the present study made use of the CFQ global severity score (*CFQ*; sum of all items; range: 0-33).

Beck Depression Inventory (BDI-II) and Beck Depression Inventory FastScreen (BDI-II-FS)

The BDI comprises 21 items. For each item, respondents have to select a response option. The symptoms severity varies between the response options and can reach a value from “0” to “3”. Higher values indicate a higher symptom severity. The total score for depression severity is the sum of severity ratings of each depression item (range 0-63). For the inclusion into the path model, we computed the seven-item *Beck Depression Inventory FastScreen (BDI-II-FS)* and which is the sum of the respective seven items that omit vegetative and somatic symptoms of depression (range: 0-21).

Patient-Determined Disease Steps (*PDDS)*

The PDDS utilizes a nine-point scale (“0” = “normal” to “8” = “bedridden”) Higher scores also indicate a higher disability.

Toronto-Alexithymia-Scale-26 (TAS-26)

The Toronto-Alexithymia-Scale-26 (*TAS-26*) is a reliable and valid tool for the assessment of alexithymia. The original version of the TAS-26 is a self-report scale and comprises 26 items. Respondents rate each item on a five-point Likert scale (“1” = “not at all true” to “5” =

"absolutely true"). The total alexithymia score is the sum of responses to 18 of the 26 items (range: 18-90).

Childhood Trauma Questionnaire (CTQ)

The present study utilized the short-version of the German CTQ [27]. This version contains 28 items. It served to assess the exposure to different types of childhood adversities. Respondents have to score each item on a five-point Likert scale ("1" = "never true"; "5" = "very often true"). Each subscale contains five items. The sum score of the items for each scale ranges from 5 to 25. Additional three items form the *denial subscale* of the questionnaire (designed to detect socially desirable responses or false-negative trauma reports). The present study did not consider the latter scale.

State-Trait Anxiety Inventory (STAI)

The State-Trait Anxiety Inventory is a widely used measure of anxiety. It contains two subscales with ten items per scale: 1. the *State Anxiety Scale* evaluates the current state of anxiety, asking how respondents feel *currently*; 2. the *Trait Anxiety Scale* evaluates relatively stable aspects of *anxiety proneness*. PwMS had to score their responses on an 8-point scale ("1" = "almost never" to "8" = "almost always"). The present study aimed to focus on the current symptoms of anxiety rather than on anxiety as a personality trait. Therefore, it utilized the State Anxiety Scale (*STAI-State*, range: 10 - 80). The German version of Grimm [29] asks not for the symptoms of anxiety at the very moment but the current symptoms. Therefore, it matched the current psychopathology in relation to the aforementioned depression and fatigue measures best.

Young Schema-Questionnaire – Short Form (YSQ)

The Young-Schema-Questionnaire utilizes a six-point Likert scale ranging from "1" = "strongly disagree" to "6" = "strongly agree". Higher values indicate a stronger presence of the respective schema. The YSQ-S3 includes 18 early maladaptive schemas (EMS), with five items per scale. In total, the YSQ-S3 consists of 90 items. In the present study, EMS belonging to a so-called domain were summed according to the five domains, postulated by Young: 1. *YSQ disconnection and rejection* (range: 25 – 150); 2. *YSQ impaired autonomy and performance* (range: 20 – 120); 3. *YSQ impaired limits* (range: 10 – 60); 4. *YSQ other-directedness* (range: 10 – 60); and 5. *YSQ over-vigilance and inhibition* (range: 20 – 120).
